# Clinical features and outcomes of COVID-19 patients with gastrointestinal symptoms

**DOI:** 10.1186/s13054-020-03034-x

**Published:** 2020-06-15

**Authors:** Chao Cao, Meiping Chen, Li He, Jiao Xie, Xiaomin Chen

**Affiliations:** 1 Department of Respiratory and Critical Care Medicine, Ningbo First Hospital, Ningbo, China; 2 Department of Respiratory and Critical Care Medicine, Jingzhou Central Hospital, the Second Clinical Medical College, Yangtze University, Jingzhou, China; 3 Department of Respiratory and Critical Care Medicine, Hubei Provincial Hospital of Integrated Chinese & Western Medicine, Wuhan, China; 4 Department of Cardiology, Ningbo First Hospital, 59 Liuting Road, Ningbo, Zhejiang province China

**Keywords:** Clinical features, Coronavirus disease 2019, Gastrointestinal symptoms

The emergence of coronavirus disease 2019 (COVID-19), which caused by severe acute respiratory syndrome coronavirus 2 (SARS-CoV-2), has put unprecedented challenges on the public health [1, 2]. It is well- known that most of the infected patients presented with fever or respiratory manifestations, while a portion of patients presented with gastrointestinal (GI) symptoms [2]. In early published study from the USA, SARS-CoV-2 viral RNA has been present in the feces of the illness [3]. However, part of COVID-19 patients present GI symptoms at the onset of diseases may be overlooked by clinicians [4].

Our experience was conducted in Ningbo First Hospital, Jingzhou Central Hospital, and Hubei Provincial Hospital of Integrated Chinese & Western Medicine. One hundred fifty-seven patients we treated were diagnosed as COVID-19 according to the World Health Organization interim guidance [5]. Nasopharyngeal swabs and chest computed tomography were collected from all patients. Demographic data, symptoms, laboratory values, comorbidities, and clinical outcomes were collected from the electronic medical records.

Of 157 patients with COVID-19, 63 (40.1%) presented with 1 or more GI symptoms (anorexia, nausea, or diarrhea). The mean age of 157 patients was 49.3 years (standard deviation, SD, 14.5), and 74 (47.1%) were male.

Of the 63 patients, 21 (33.3%) had nausea, 47 (74.6%) had anorexia, and 25 (39.7%) had diarrhea. The mean age of those patients was 51.9 years (SD, 14.9). Twenty-four (38.1%) were male, and 24 (38.1%) had chronic diseases. The most common symptoms were cough, fatigue, fever, and muscle soreness. Neither the median white blood cell nor lymphocyte counts were different between patients with and without GI symptoms (Table 1).

**Table 1:** Demographics and clinical features of coronavirus disease 2019

	Total (***n*** = 157)	GI symptoms(***n*** = 63)	Without GI symptoms (***n*** = 94)	***p*** value*
**Age, mean (SD), years**	49.3 (14.5)	51.9 (14.9)	47.5 (14.0)	0.0599
**Gender**				
Male	74 (47.1%)	24 (38.1%)	50 (53.2%)	0.0633
Female	83 (52.9%)	39 (61.9%)	44 (46.8%)	
**Comorbidities**				
Hypertension	28 (17.8%)	12 (19.1%)	16 (17.0%)	0.7451
Diabetes	9 (5.7%)	5 (7.9%)	4 (4.3%)	0.5337
Chronic kidney disease	3 (1.9%)	1 (1.6%)	2 (2.1%)	1.0000
Chronic lung disease	2 (1.3%)	1 (1.6%)	1 (1.1%)	1.0000
Heart disease	2 (1.3%)	2 (3.2%)	0	0.1595
Malignancy	4 (2.6%)	1 (1.6%)	3 (3.2%)	0.9135
Total with ≥ 1 comorbidity	55 (35.0%)	24 (38.1%)	31 (33.0%)	0.5101
**Symptoms**				
Fever	65 (41.4%)	23 (36.5%)	42 (44.7%)	0.3082
Cough	109 (69.4%)	47 (74.6%)	62 (66.0%)	0.2491
Sore throat	12 (7.6%)	4 (6.4%)	8 (8.5%)	0.8468
Muscle soreness	44 (28.0%)	23 (36.5%)	21 (22.3%)	0.0527
Fatigue	73 (46.5%)	44 (69.8%)	29 (30.9%)	< 0.001
**Initial laboratory parameters, median (IQR)**				
WBCs count, × 10^9^/L	4.9 (3.8–6.3)	4.9 (3.4–6.0)	5.0 (4.0–6.4)	0.4838
Lymphocyte count, × 10^9^/L	1.0 (0.7–1.4)	1.0 (0.7–1.4)	1.0 (0.7–1.5)	0.4423
C-reactive protein, mg/L	13.2 (3.4–32.9)	17.8 (7.2–41.1)	9.1 (2.9–30.3)	0.0561
ALT level, IU/L	21.7 (15.4–38.8)	23.1 (15.0–43.0)	21.7 (16.2–34.3)	0.8062
AST level, IU/L	26.2 (20.7–34.7)	26.0 (20.0–35.0)	26.9 (20.8–34.7)	0.7189
**Severe cases**	41 (26.1%)	8 (12.7%)	33 (35.1%)	0.0016
**Corticosteroid usage**	112 (71.3%)	40 (63.5%)	72 (76.6%)	0.0751
**Hospital course, mean (SD), days**				
Duration onset to treatment	5.3 (5.4)	5.9 (6.0)	4.9 (4.9)	0.2580
Clinical recovery time	9.8 (4.9)	10.7 (4.5)	9.1 (5.2)	0.0607
Time of virus nucleic acid turn to negative	12.4 (6.4)	13.0 (6.1)	12.0 (6.7)	0.3509
Hospitalization duration	16.0 (4.9)	16.1 (5.1)	15.8 (4.7)	0.7003

*GI* gastrointestinal, *IQR* interquartile range, *SD* standard deviation, *WBC* white blood cell, *ALT* alanine aminotransferase, *AST* aspartate aminotransferase

*P values indicate differences between patients with GI symptoms and those without. *P* < 0.05 was defined as statistically significant

There was no significant difference in viral shedding, the time to clinical recovery, or hospitalization duration between patients with and without GI symptoms (Table 1). Among patients with GI symptoms, 63.5% received corticosteroids treatment, which is much lower than patients without GI symptoms group (63.5% vs 76.6%; *p* = 0.0751). Moreover, less patients with GI symptoms developed into severe cases compared with those without GI symptoms (12.7% vs 35.1%; *p =* 0.0016).

In our experience, 4 out of 10 patients with COVID-19 have significant GI symptoms. There was no significant difference in gender, age, and comorbidities between patients with and without GI symptoms. Leukocyte and lymphocyte counts were similar between the two groups. Besides, there was no significant difference in viral shedding, the time to clinical recovery, or hospitalization duration between patients with and without GI symptoms. Nonetheless, less patients with GI symptoms received corticosteroids and developed into severe cases.

This study suggested that GI symptoms in COVID-19 are frequent but are not associated with the severity of diseases or worse outcomes. However, because SARS-CoV-2 can be found in patient feces and the digestive system, we should be cautious with these potential routes for transmission [2, 3]. This study is limited by the lacked of data of reverse transcriptase polymerase chain reaction on COVID-19 in GI specimens. Our observations indicate that a substantial number of patients present with predominantly GI symptoms, and caution about this atypical presentation is necessary.

## Data Availability

Participant data without names and identifiers will be made available after approval from the corresponding author.

## Abbreviations

COVID-19: Coronavirus disease 2019
SARS-CoV-2: Severe acute respiratory syndrome coronavirus 2
GI: Gastrointestinal
IQR: Interquartile range
SD: Standard deviation
WBC: White blood cell
ALT: Alanine aminotransferase
AST: Aspartate aminotransferase
